# Supplementary material for: Spatial transcriptomics landscape of lesions from non-communicable inflammatory skin diseases
Source: Nat Commun. 2022 Dec 13;13:7729. doi: 10.1038/s41467-022-35319-w (PMC9747967; doi:10.1038/s41467-022-35319-w)
Supplement: Supplementary file 1 — Supplementary Information [file 41467_2022_35319_MOESM1_ESM.pdf]

Supplementary Information

Supplementary Figures

Supplementary Figure 1

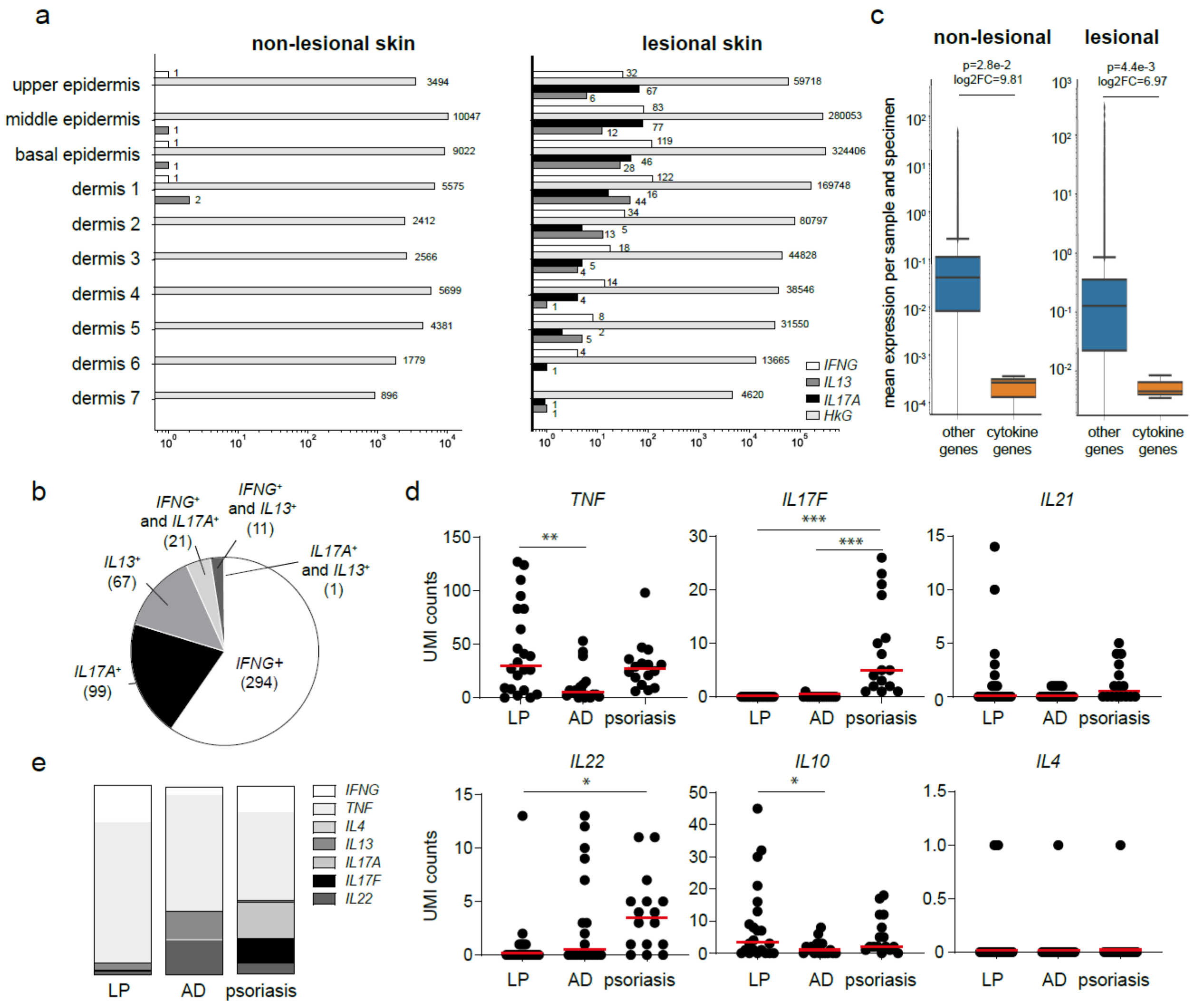

Supplementary Figure 1: NcISD are characterized by low cytokine UMI counts in skin
a) Total UMI counts in spatial sections for *IFNG, IL17A, IL13* and the housekeeping gene *GAPDH* (*HkG*) in non-lesional and lesional skin separated by the location in the skin. **b)** Percentage of cytokine single and double-transcript positive cells. **c)** Mean expression per sample and specimen of cytokine (*IFNG, IL13, IL17A*) (orange) and all other genes (blue) in non-lesional (n=14) and lesional skin (n=31). Two-sided Mann-Whitney test was used as a statistical test. (non-lesional (others; cytokines): median (0; 0), mean (1.82e-1; 2.02e-4), min (0; 0), max (3e-1; 0), Q1(0;0), Q3 (1e-1; 0), whiskers (0; 1e-1); lesional (others; cytokines): median (1e-1; 0), mean (6.87e-1; 5.49e-3), min (0; 0), max (8e-1; 0), Q1(0;0), Q3 (4e-1; 0), whiskers (0; 4e-1) **d)** UMI counts for selected cytokines in sections of LP (n=22), atopic dermatitis (AD (n=18), and psoriasis (n=18). Statistical significance was determined using One-Way Anova and Turkey's multiple

comparisons test without FDR correction. *<0.05, **<0.01, ***<0.001. **e)** Percentage of disease relevant cytokine UMI counts in LP, AD, and psoriasis normalised to 100%.

Supplementary Figure 2

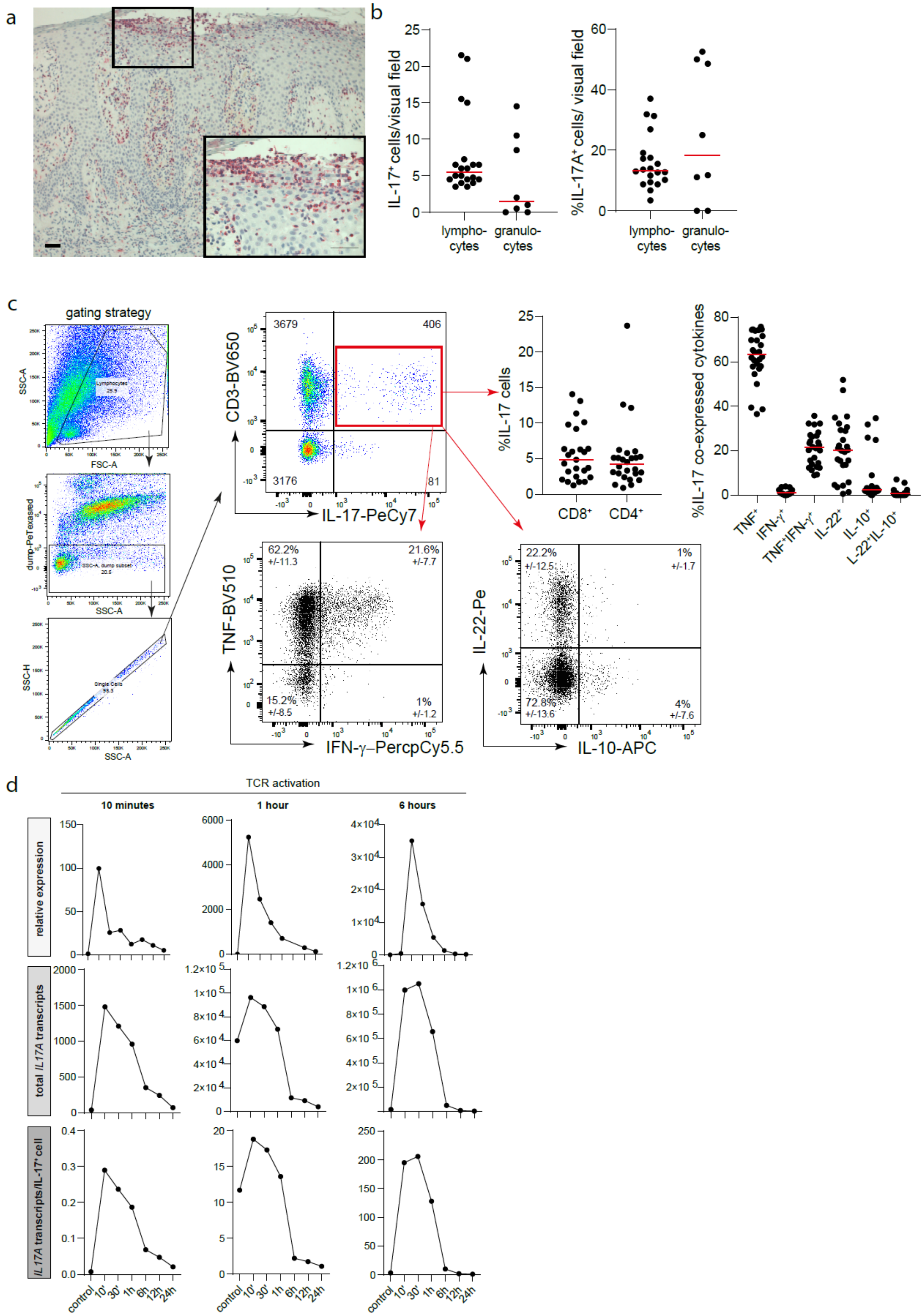

Supplementary Figure 2: IL-17A expression in skin lesions and infiltrating T cells and its short half-life time and low copy numbers in in vitro stimulated T cells

a) Representative staining of IL-17A by immunohistochemistry in a psoriasis section. Scale bar: 20µM **b)** Number (left panel) and percentage (right panel) of IL-17A$^+$ lymphocytes and granulocytes per visual field in psoriasis sections stained by immunohistochemistry (n=20 patients). **c)** Gating strategy and representative flow cytometry staining of CD3$^+$IL-17$^+$ T cells derived from lesional psoriasis skin. CD3$^+$IL-17$^+$ were gated to analyse co-production of IL-17A with IL-22, TNF, IL-10, and IFN-**γ** by intracellular flow cytometry. The graphs indicate the percentage of CD4$^+$ and CD8$^+$ cells amongst the CD3$^+$IL-17A$^+$ cells and the frequency of IL-17A producing cells co-expressing one or two other cytokines (n=52). **d)** CD4+ T cells were isolated from blood of healthy donors and stimulated with anti-CD3/anti-CD28 antibodies (TCR activation) for the indicated time. RNA was isolated over a time course of 24 h and analysed for the expression of *IL17A* by real time PCR. Relative expression of *IL17A* was calculated to unstimulated cells (upper panel). Total transcript numbers of *IL17A* were determined in each stimulatory condition using a standard curve (middle panel). By dividing the total transcript numbers by the number of cells per stimulatory conditions, the transcript number per cell could be identified (lower panel).

Supplementary Figure 3

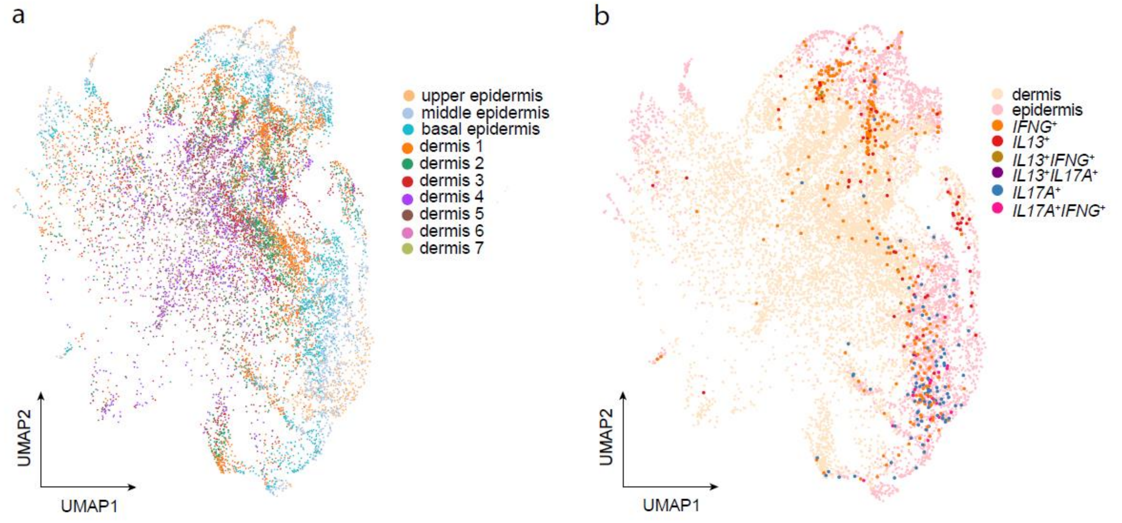

Supplementary Figure 3: Cytokine transcript-positive spots are located in the epidermis and enriched in lesional skin

a) UMAP plot highlighting the manually annotated tissue layers basal, middle and upper epidermis and dermis depth 1-7 in all spatial samples expressing leukocyte markers. **b)** UMAP plot indicating cytokine transcript-positive spots in epidermis and dermis in spatial sections expressing leukocyte markers.

Supplementary Figure 4

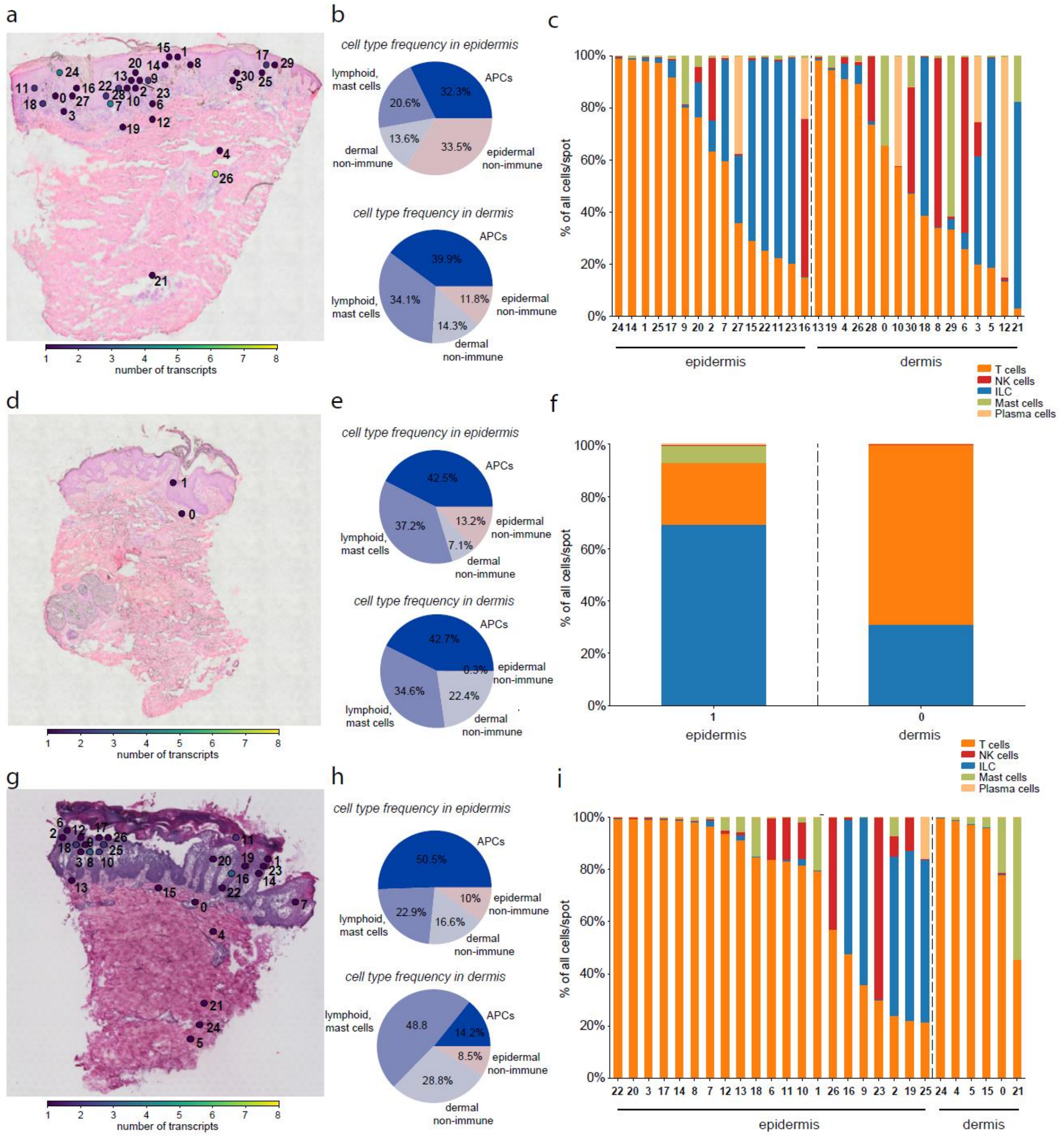

Supplementary Figure 4: Tangram analysis of cytokine-transcript positive spatial spots reveals heterogeneous cellular composition

Numbering of *IFNG* (**a**), *IL13* (**d**) and *IL17A* (**g**) transcript positive leukocyte spots in lichen planus, atopic dermatitis and psoriasis ST sections, respectively. The scale indicates the number of transcripts detected in each spot. Cellular composition of *IFNG* (**b**), *IL13* (**e**) and *IL17A* (**h**) transcript-positive spatial spots in epidermis and dermis. Epidermal non-immune cells: keratinocytes and melanocytes; dermal non-immune cells: fibroblasts, pericytes, Schwann cells, vascular and lymphatic endothelial cells. APC: dendritic cells, monocyte-derived dendritic cells, Langerhans cells, macrophages, inflammatory macrophages, monocyte-

derived macrophages. Zoom into the lymphoid and mast cell composition of *IFNG* (**c**), *IL13* (**f**) and *IL17A* (**i**) transcript-positive spatial spots in epidermis and dermis.

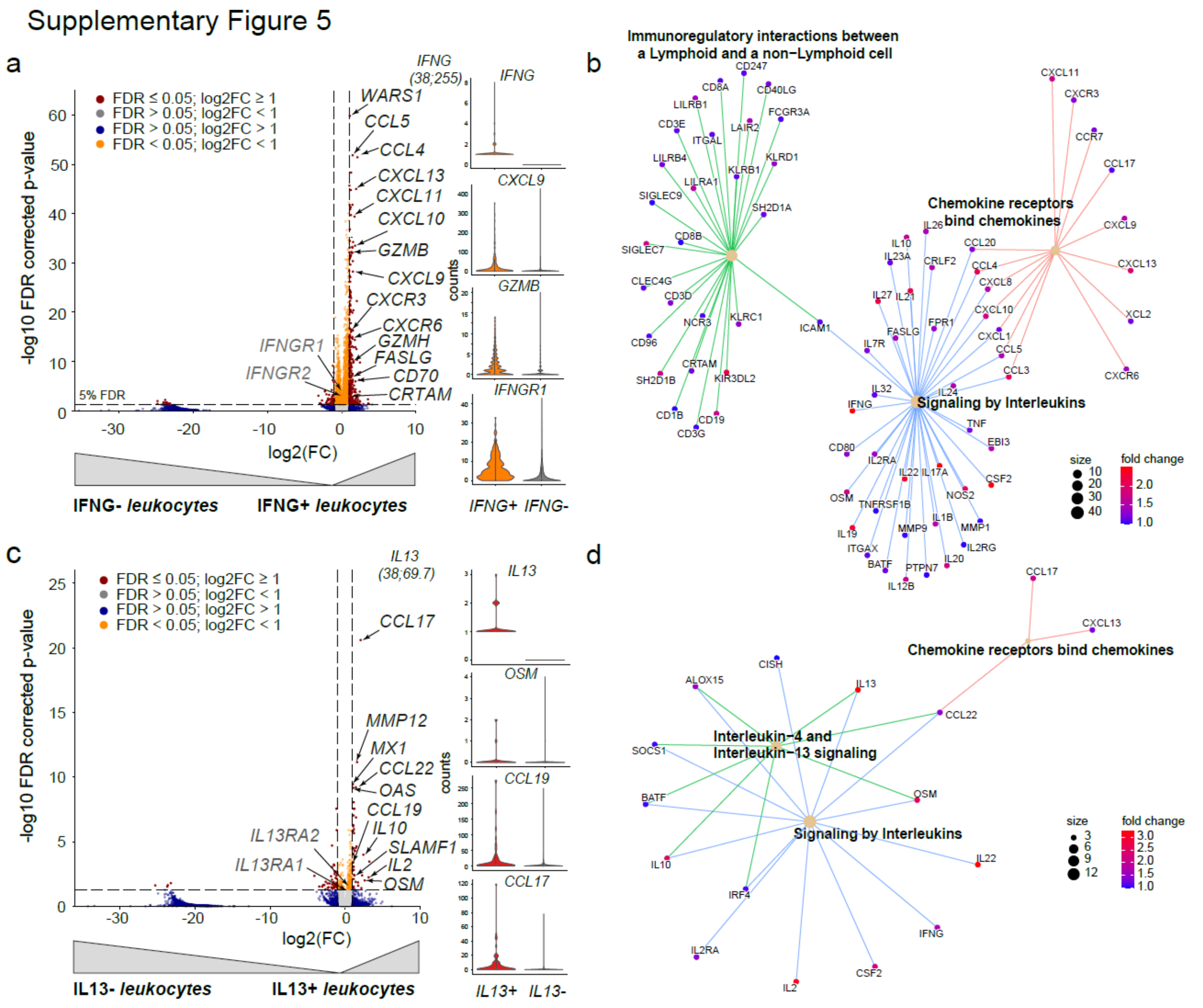

Supplementary Figure 5: Cytokine transcript-positive leukocyte spots are characterized by specific gene signatures

a) Volcano plot analysing the gene expression profile of *IFNG* transcript-positive (*IFNG*$^{+}$) *versus IFNG transcript*-negative (*IFNG*$^{-}$) leukocyte spots in ST sections (n=82). Coordinates for *IFNG* (38/255) are not shown. Violin plots show expression of selected genes in both groups. **b)** Gene set enrichment analysis of genes co-expressed with *IFNG.* **c)** Volcano plot analysing the gene expression profile of *IL13 transcript*-positive (*IL13*$^{+}$) *versus IL13 transcript*-negative (*IL13*$^{-}$) leukocyte spots. Coordinates for *IL13* (38.1/69.7) are not shown. Violin plots show expression of selected genes in both groups. **d)** Gene set enrichment analysis of genes co-expressed with *IL13*. Benjamini Hochberg was used to determine statistical significance in a) and c).

Supplementary Figure 6

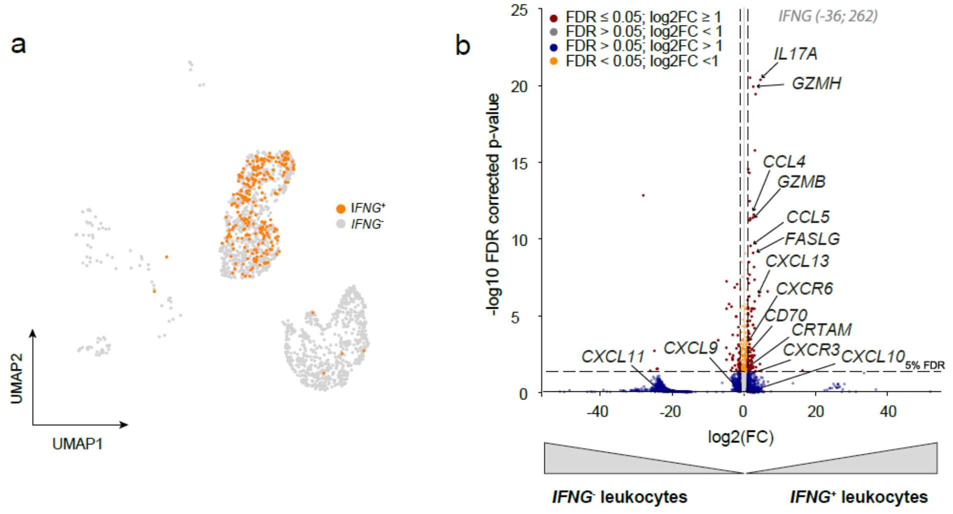

Supplementary Figure 6: Single-cell analysis reveals specific gene signatures for IL17A and IFNG expressing cells

a) UMAP plot highlighting *IFNG* transcript-positive (*IFNG*$^{+}$) leukocytes in orange and *IFNG* negative (*IFNG*$^{-}$) leukocytes in grey. **b)** Volcano plot analysing differentially expressed genes (DEG) *IFNG* transcript-positive (*IFNG*$^{+}$*) versus IFNG* transcript-negative (*IFNG*$^{-}$) leukocytes in the single-cell data set. Coordinates of *IFNG (*-36/262) are not shown. Benjamini Hochberg was used to determine statistical significance.

Supplementary Figure 7

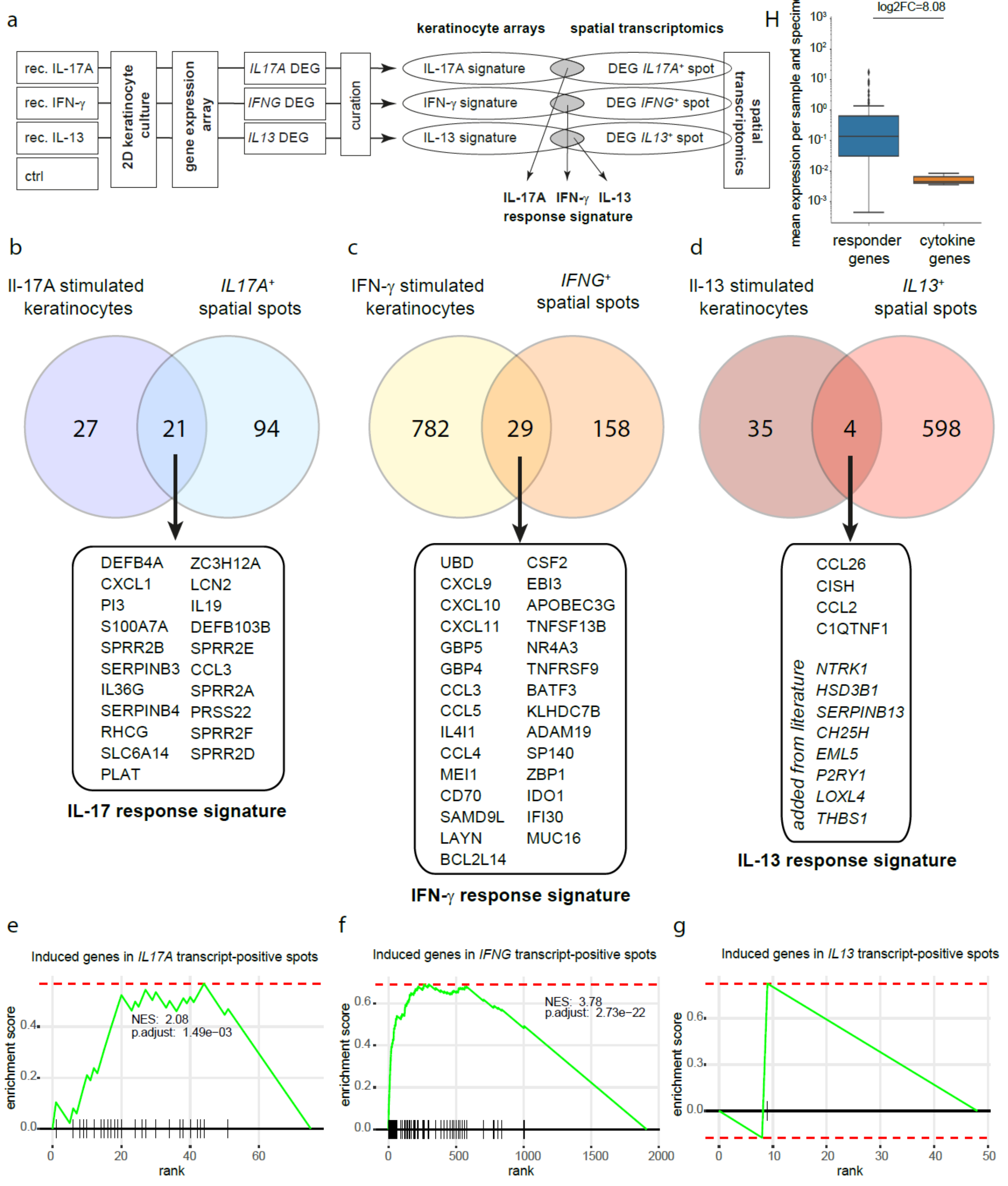

Supplementary Figure 7: Identification of cytokine responder genes for spatial correlation

a) Primary human keratinocytes were stimulated in 2D cultures with recombinant IL-17A, IFN-**γ**, or IL-13 (20 ng/ml each) for 16 h. Total RNA was isolated and whole genome expression arrays (SurePrint G3 Human GE 8X60K BeadChip (Agilent Technologies)) were performed according to the manufacturer's instructions. Gene expression data was filtered for p-value <0.05, adjusted p-value <0.05 and log2 FC >1.5

for IL-17A and IFN-γ or log2FC >1 for IL-13. Differential genes co-expressed in cytokine stimulated keratinocytes and cytokine transcript-positive spatial spots were determined for IL17-A, IFN-g, and IL-13 and overlapping genes were defined as response signature for each cytokine. Specific response signatures for **b)** IL17-A, **c)** IFN-g and **d)** IL-13. For *IL13* only 4 genes were commonly expressed, therefore, additional genes were added according to literature analysis. Enrichment analysis of response signature genes for **e)** *IL17A*, **f)** *IFNG,* and **g)** *IL13* (black bars) within the DEGs of the respective cytokine transcript-positive spots. **h**) Mean expression per sample and specimen of cytokines (*IFNG, IL13, IL17A*) and their specific responder genes in lesional skin samples (n=31). Responder genes are depicted in blue, cytokine genes in orange. Two-sided Mann-Whitney test was used to calculate the p-value. (responder genes; cytokine genes): median (1e-1; 0), mean (1.48; 5.49e-3), min (0; 0), max (1.3; 0), Q1(0;0), Q3 (46e-1; 0), whiskers (0; 46e-1).

Supplementary Figure 8

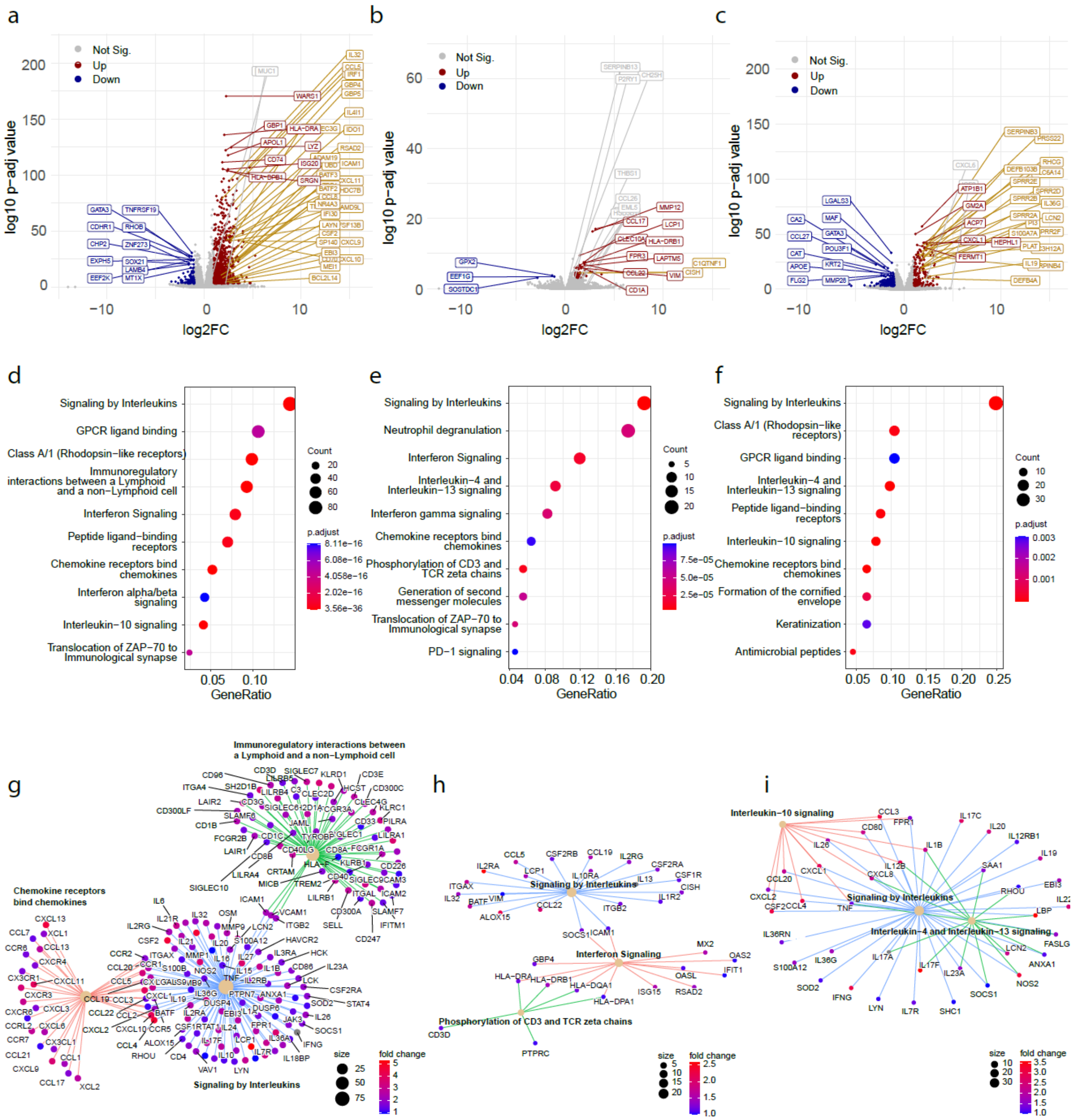

Supplementary Figure 8: Spatial transcriptomics enables to identify new cytokine-related responder genes and thereby potential drug targets

a-c) To identify potentially new cytokine-related responder genes, differentially expressed genes (DEG) between the optimal radius of each cytokine (*IFNG* radius 4 (**a**); *IL13* radius 3 (**b**) and *IL17A* radius 0 (**c**)) and cytokine transcript-negative spots outside this optimal radius were determined and presented as a zoomed-in Volcano plot). Experimentally identified responder genes (see Fig. S7) (gold (p-adj <0.05, abs(log2FC) >1) or grey (p-adj >0.05)) and new, data-derived cytokine associated genes (up-regulated in blue; down-regulated in red) are shown. **d-g)** Reactome pathways for *IFNG*-related (**d**), *IL13*-related (**e**) and *IL17A*-related (**f**) genes as shown in Fig. S8 A-C (padj <0.05, abs(log2FC) >1). **g-i)** Pathway enrichment

analysis of top three pathways for *IFNG*-related (**g**), *IL13*-related (**h**) and *IL17A*-related (**i**) genes (p-adj <0.05, abs(log2FC) >1). Benjamini Hochberg was used to determine statistical significance in a-f).

Supplementary Tables

	sex	n	age	severity score
**Psoriasis**	m	7	41,14 ± 14,74	PASI: 11,76 ± 4,97
	w	4	56,75 ± 9,54	PASI: 9,95 ± 3,74
**Atopic dermatitis**	m	8	49,38 ± 7,48	EASI (n=4): 22,35 ± 11,75<br>SCORAD (n=2): 58 ± 9
	w	1	21	56
**Lichen planus**	m	6	40,67 ± 10,39	*no score available*
	w	5	56,6 ± 7,8	*no score available*

Supplementary Table 1: *Patient characteristics of the spatial transcriptomics cohort*

Disease	Patient	slide number	non-lesional (nl) lesional (l)	Number of spots/section	Total UMI count/section	Median UMI count/spot/section	Number IFNg+ spots/section	Median IFNg UMI count/IFNg+ spot	Number IL13+ spots/section	Median IL-13 UMI count/IL-13+ spot	Number IL17+ spots/section	Median IL-17 UMI count/IL-17+ spot
AD	2	2-V19S23-004-V1_2	nl	587	3332849	920	0	0	0	0	0	0
		2-V19S23-004-V2_2	nl	464	3758790	1239	0	0	0	0	0	0
		2-V19S23-004-V3_2	l	527	7298036	1305	0	0	2	1	0	0
		2-V19S23-004-V4_2	l	524	11691137	4506,5	0	0	0	0	0	0
	5	5-V19S18-093-V1_5	nl	525	1050295	98	0	0	0	0	0	0
		5-V19S18-093-V2_5	nl	768	1865251	151	0	0	0	0	0	0
		5-V19S18-093-V3_5	l	442	2299813	803	0	0	0	0	0	0
		5-V19S18-093-V4_5	l	469	1861982	477	0	0	0	0	0	0
	8	8-V19T12-006-V1_8	nl	698	4526990	382,5	0	0	3	1	0	0
		8-V19T12-006-V2_8	nl	765	1120717	325	0	0	0	0	0	0
		8-V19T12-006-V3_8	l	697	2062754	2113	1	1	1	1	0	0
		8-V19T12-006-V4_8	l	706	8743365	2524	1	1	3	1	0	0
	11	11-V19T12-012-V1_11	nl	305	1443493	2063	2	1	0	0	0	0
		11-V19T12-012-V2_11	nl	545	2146796	861	0	0	1	1	0	0
		11-V19T12-012-V3_11	l	713	3519737	494	0	0	1	1	0	0
		11-V19T12-012-V4_11	l	923	3765271	619	0	0	0	0	0	0
	15	15-V19S18-092-V1_15	l	1350	4945612	45	2	1	2	1	0	0
		15-V19S18-092-V2_15	l	1504	5490636	84	1	1	1	2	0	0
	20	SN-V11J13-122_A_20	l	181	2018930	2115	1	1	1	1	0	0
		SN-V11J13-122_B_20	l	727	4756457	1803	1	1	3	1	0	0
	34	SN-V11J13-122_A_34	l	248	15579630	40857,5	1	1	10	1	0	0
		SN-V11J13-122_B_34	l	358	16844100	25051,5	2	1	11	1	0	0
	35	SN-V11J13-122_C_35	l	995	8778245	1346	1	1	2	1	1	1
		SN-V11J13-122_D_35	l	986	9860581	1278	4	1	6	1	0	0
	36	SN-V11J13-122_C_36	l	102	632857	235	0	0	0	0	0	0
		SN-V11J13-122_D_36	l	47	694052	325	0	0	0	0	0	0
LP	3	3-V19S23-005-V1_3	nl	546	1148998	746,5	0	0	0	0	0	0
		3-V19S23-005-V2_3	nl	549	1032311	667	1	1	0	0	0	0
		3-V19S23-005-V3_3	l	450	7837183	2097	1	1	2	1	0	0
		3-V19S23-005-V4_3	l	825	14444608	4877	16	1	4	1	0	0
	6	6-V19T12-047-V1_6	nl	666	1232549	189,5	0	0	0	0	0	0
		6-V19T12-047-V2_6	nl	706	784663	240	0	0	0	0	0	0
		6-V19T12-047-V3_6	l	1000	15217246	1137	37	1	7	1	2	1
		6-V19T12-047-V4_6	l	985	14144635	1965	31	1	6	1	0	0
	9	9-V19T12-015-V1_9	nl	508	3532882	2077,5	0	0	0	0	0	0
		9-V19T12-015-V2_9	nl	566	3094003	1740,5	0	0	0	0	0	0
		9-V19T12-015-V3_9	l	814	9496593	2378,5	15	1	1	1	0	0
		9-V19T12-015-V4_9	l	913	9264034	2570	19	1	2	1	0	0
	12	12-V19T12-021-V1_12	nl	625	1438737	696	0	0	0	0	0	0
		12-V19T12-021-V2_12	nl	651	1729572	679	0	0	0	0	0	0
		12-V19T12-021-V3_12	l	695	5987919	1159	8	1	1	1	0	0
		12-V19T12-021-V4_12	l	1040	8870277	1496,5	30	1	12	1	0	0
	14	14-V19T12-024-V1_14	nl	598	738422	38,5	0	0	0	0	0	0
		14-V19T12-024-V2_14	nl	744	422422	59	0	0	0	0	0	0
		14-V19T12-024-V3_14	l	1122	13046660	223,5	6	1	0	0	3	2
		14-V19T12-024-V4_14	l	1117	8378001	127	2	1	0	0	0	0
	25	SN-V11J13-122_A_25	l	307	8348512	20988	1	1	0	0	0	0
		SN-V11J13-122_B_25	l	1428	19259028	6320	20	1	1	1	0	0
	26	SN-V11J13-120_A_26	l	1222	577953	206,5	1	1	0	0	0	0
		SN-V11J13-120_B_26	l	1169	1162702	302	0	0	1	1	0	0
	27	SN-V11J13-120_C_27	l	727	542865	187	0	0	0	0	0	0
		SN-V11J13-120_D_27	l	810	881062	410	0	0	0	0	0	0
	28	SN-V11J13-119_C_28	l	971	2082618	659	4	1	0	0	0	0
		SN-V11J13-119_D_28	l	1064	14142627	5820,5	4	1	1	1	1	1
	30	SN-V11J13-120_A_30	l	729	604015	596	9	1	6	1	0	0
		SN-V11J13-120_B_30	l	898	1372578	780	3	1	2	1	0	0
	37	SN-V11J13-120_C_37	l	627	423276	411	1	1	0	0	0	0
		SN-V11J13-120_D_37	l	584	481369	465	2	1	1	1	0	0
Pso	1	V19523-003-V1_1	nl	818	719053	243	0	0	0	0	0	0
		V19523-003-V2_1	nl	777	304605	41	0	0	0	0	0	0
		V19523-003-V3_1	l	909	7325920	242	20	1	0	0	16	1
		V19523-003-V4_1	l	752	8024328	1574,5	15	1	0	0	27	1
	10	10-V19T12-025-V1_10	nl	617	446231	192	0	0	0	0	0	0
		10-V19T12-025-V2_10	nl	585	679701	151	0	0	0	0	0	0
		10-V19T12-025-V3_10	l	1021	5873368	552	4	1	0	0	4	1
		10-V19T12-025-V4_10	l	1014	4245034	539	3	1	0	0	8	1
	13	13-V19T12-048-V1_13	nl	790	2225142	452,5	0	0	0	0	0	0
		13-V19T12-048-V2_13	nl	791	1563868	287	0	0	0	0	0	0
		13-V19T12-048-V3_13	l	1144	4970420	297,5	2	1	2	1	7	1
		13-V19T12-048-V4_13	l	1414	6686569	502	2	1	0	0	3	1
	19	SN-V10N16-107_A_19	l	1079	15269051	1983	0	0	0	0	5	1
		SN-V10N16-107_B_19	l	997	15906332	3798	4	1	0	0	2	1
	22	SN-V10N16-107_A_22	l	885	13980294	3050	15	1	0	0	15	1
		SN-V10N16-107_B_22	l	909	13551685	2874	11	1	0	0	12	1
	29	SN-V10N16-107_C_29	l	691	2311770	889	2	1	0	0	3	1
		SN-V10N16-107_D_29	l	736	1600795	956,5	2	1	1	1	2	1,5
	31	SN-V11J13-119_A_31	l	1276	23903564	3004,5	6	1	0	0	7	1
		SN-V11J13-119_B_31	l	696	15673596	4435	3	1	0	0	4	1
	32	SN-V11J13-119_A_32	l	703	10738392	2694	4	2,5	1	1	7	1
		SN-V11J13-119_B_32	l	557	14396251	6936	8	1	4	1	5	2
	33	SN-V11J13-119_C_33	l	1072	8954290	2530	15	1	0	0	12	1
		SN-V11J13-119_D_33	l	923	26657774	6991	28	1	1	1	8	1

Supplementary Table 2: *Overview on number of spots, UMI counts and cytokine transcript-positive spots in the ST dataset*
